# Optically Tunable Gratings Based on Coherent Population Oscillation

**DOI:** 10.1038/s41598-018-25010-w

**Published:** 2018-05-01

**Authors:** Xiao-Jun Zhang, Hai-Hua Wang, Lei Wang, Jin-Hui Wu

**Affiliations:** 1 Center for Quantum Sciences, Northeast Normal University, Jingyue Street 2555, Changchun, 130117 China; 2 Changchun Observatory, National Astronomical Observatories, CAS, Changchun, 130117 China; 3 College of Physics, Jilin University, Changchun, 130023 China

## Abstract

We theoretically study the optically tunable gratings based on a L-type atomic medium using coherent population oscillations from the angle of reflection and transmission of the probe field. Adopting a standing-wave driving field, the refractive index of the medium as well as the absorption are periodically modified. Consequently, the Bragg scattering causes the effective reflection. We show that different intensities of the control field lead to three types of reflection profile which actually correspond to different absorption/amplification features of the medium. We present a detailed analyses about the influence of amplification on the reflection profile as well. The coherent population oscillation is robust to the dephasing effect, and such induced gratings could have promising applications in nonlinear optics and all-optical information processing.

## Introduction

Periodic optical media have attracted a great deal of attentions. One instance is the photonic crystals whose refractive index varies periodically over a length scale comparable to optical wavelength. Fascinating effects of photonic crystals have been examined in the field of quantum optics, such as controlling spontaneous and thermal emission^1–3^, efficient miniature laser^4^, controlling interaction between individual photons^5^, and efficient photonelectric conversion in solar cell^6,7^, just to mention a few. Conventional photonic crystals, for example, the solid materials with arranged air holes^8^, silicon woodpile^9^, and honeycomb structures^10^ and so on, have fixed configurations. The photonic band structures of such materials are determined. And clearly, the tunable photonic crystal has more promising applications. One approach to produce such material is to introduce standing-wave field into the interaction between light and matters, and use the standing-wave field to periodically modify the refractive index of the sample, and further to control the optical properties of the constructed periodic structure. Such tunable structure is often refered to as electromagnetically induced grating (EIG)^11^. And it has important applications in quantum memory^12,13^, enhanced nonlinear optics process^14,15^, and deeply relates to coherent control of light propagation^16,17^, and photon state manipulation^18^. Electromagnetically induced transparency (EIT)^19^ is the common choice to establish EIG. With a strong coupling field and a weak probe field interacting with the atomic media, normally in a three-level Λ configuration, EIT medium can efficiently limit the absorption of the resonant probe field due to the atomic coherence built between two ground states. The feature of EIT is a narrow transparency window in the absorption profile, with the width that can be controlled by the Rabi frequency of the coupling field^20^. Adopting a standing-wave field as the coupling field, the refractive index and the absorption are periodically modified, then EIG is constructed^21^.

There are other kinds of optical tunable gratings, for example, the four-wave mixing gratings^22,23^ which reduce the power threshold for generating the optical phase-conjugate beam, and the Raman-induce gratings^17^ with the probe field operating in a stimulated Raman emission mode to eliminate the signal attenuation^24^. However, the gratings we mentioned above essentially relates to the atomic coherence between the ground states^24–26^, and are vulnerable to dephasing effect. Such characteristic is experimentally demonstrated in the example of EIT system with the dephasing effect represented by the gradient of magnetic field^27,28^.

In the present paper, we propose a novel scheme to construct optically tunable gratings based on coherent population oscillations (CPOs)^29,30^. Normally CPO can be realized by applying two coherent electromagnetic fields of different amplitudes and frequencies onto a two-level atomic system with an additional shelving state. This system has potential application in spatial optical memory. An alternative avenue is to employ the Λ-system^27,28^ (adopted in our present investigation) composed of two coupled two-level subsystem. Such type of CPO is characterized by a narrow transparency window with the width depending on the population decay rate between the two ground states. Using a standing-wave driving field as the control field, the CPO-induced grating can be constructed. Such gratings are robust to dephasing effect since atomic coherence is not involved^31^, and possible gain can be introduced to overcome the absorption of the probe field. In the following sections we systematically investigate it from the point of view of reflection and transmission. The equations describing the CPO system are given in Sec. II together with the introduction of the mechanism. In Sec. III we investigate the properties of the gratings using the characteristic-matrix method. And finally a brief conclusion is present in Sec. IV.

## Basic Equations

**Figure 1:**
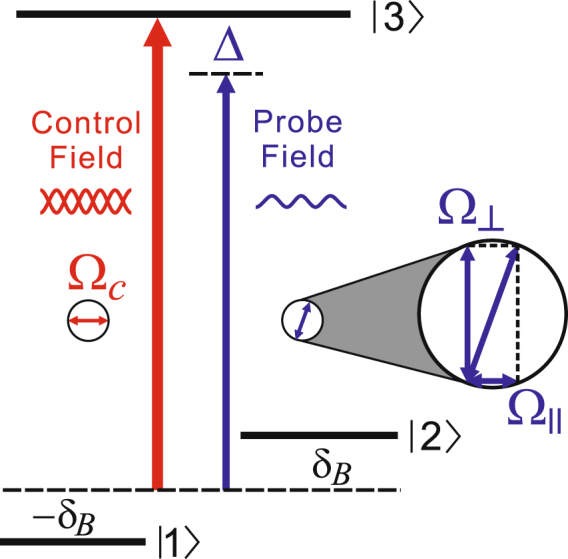
Schematic diagram of linearly polarized (represented by the double-headed arrow in circle) control field and probe field interacting with the three-level atomic system.

Let us consider the atomic system shown in Fig. 1 with the optical transition frequency represented by *ω*_0_. And the magnetic quantum numbers of the states |1〉, |2〉 and |3〉 are *m* = −1,1, and 0 respectively. In other words, the transition |1〉−|3〉 (|2〉−|3〉) can be coupled by the *σ*_+_ (*σ*_−_) polarized light. The ground states are shifted by 2*δ*_*B*_ due to the Zeeman effect of a magnetic field **B**. The commonly used medium for demonstrating CPO is metastable helium^28,32^ which is adopted in our investigation as well. A strong standing wave *E*_*c*_ resonant with the optical transition serves as the control field, and a weak field *E*_*p*_ with frequency *ω*_*c*_−Δ probes the three-level system. Here *E*_*c*_ (*E*_*p*_) is the amplitude of the corresponding field. The control field is linearly polarized, then without loss of generality, we set its two circularly-polarized components in phase, ${{\rm{\Omega }}}_{c}=\frac{1}{\sqrt{2}}({{\rm{\Omega }}}_{c+}+{{\rm{\Omega }}}_{c-})$. Here Ω_*c*_ and Ω_*c*±_ defined as ${{\rm{\Omega }}}_{c,c\pm }=\wp {E}_{c,c\pm }/2\hslash$ stand for the Rabi frequencies of the control field, and its *σ*_±_-components respectively. The dipole moments of the two optical transitions having the same value $\wp$ is already assumed. Taking the polarization of the control field as the reference, the linearly-polarized component the probe field parallel to the control-field polarization is represented by ${{\rm{\Omega }}}_{\parallel }$, while the perpendicular one, by Ω_⊥_. Adopting the relations ${{\rm{\Omega }}}_{+}=({{\rm{\Omega }}}_{\parallel }+i{{\rm{\Omega }}}_{\perp })/\sqrt{2}$, ${{\rm{\Omega }}}_{-}=({{\rm{\Omega }}}_{\parallel }-i{{\rm{\Omega }}}_{\perp })/\sqrt{2}$, where Ω _+_ and Ω_−_ stand for the two circularly-polarized components of the probe field, we can write the Hamiltonian of the three-level atom in a rotating frame as

1 $$H=-\,\hslash (\begin{array}{ccc}0 & 0 & {{\rm{\Omega }}}_{1}^{\ast }\\ 0 & -2{\delta }_{B} & {{\rm{\Omega }}}_{2}^{\ast }\\ {{\rm{\Omega }}}_{1} & {{\rm{\Omega }}}_{2} & -{\delta }_{B}\end{array}).$$

Where ${{\rm{\Omega }}}_{1}={{\rm{\Omega }}}_{c+}+{{\rm{\Omega }}}_{+}{e}^{i{\rm{\Delta }}t}$, ${{\rm{\Omega }}}_{2}={{\rm{\Omega }}}_{c-}+{{\rm{\Omega }}}_{-}{e}^{i{\rm{\Delta }}t}$. The dynamics of laser-driven atomic system is governed by the density matrix *ρ*, which obeys the master equation *iħ*∂_*t*_*ρ* = [*H*,*ρ*]. The solution to the above partial differential equation for the *resonant* probe field is discussed in the supplemental material of the article^31^. Take it as a major reference, solving the equations with the non-zero Δ is straightforward using the method of Floquet expansion^29,33,34^.

Regarding that Ω_±_ are small values as compared with Ω_*c*±_. The above equations can be solved using the perturbation method with respect to Ω_±_. Note that the Raman coherence *ρ*_12_ (*ρ*_21_) should be neglected in every order since the Zeeman shift is large enough to avoid it to be built up^28,31,32^. We further assume that Ω_*c*±_ can be described by real numbers, ${{\rm{\Omega }}}_{c+}={{\rm{\Omega }}}_{c-}=\frac{1}{\sqrt{2}}{{\rm{\Omega }}}_{c}$, ${{\rm{\Omega }}}_{c}\in {\mathbb{R}}$. The zero-order equations and their steady-state solutions where only the control field is considered and the first-order equations with the effect of the probe field included are presented in the Supplementary Information. The relevant density matrix ${\rho }_{ij}^{(1)}$ has been expanded into series as ${\rho }_{ij}^{(1)}={\sum }_{n}{\rho }_{ij}^{ < n > }{e}^{in{\rm{\Delta }}t}$. Note that we use the terms in round bracket (·) in the superscript to represent the levels of the perturbation with respect to Ω_±_, and the terms in angle bracket <·> to distinguish the coefficients in the expansion. In this system, for example, ${\rho }_{31}^{ < 1 > }$ relates to the atomic polarization excited by the *σ*_+_ -component of the probe field whose frequency is *ω*_0_ − Δ, meanwhile ${\rho }_{31}^{ < -1 > }$ corresponds to the generation of an idle field^29^ with the frequency of *ω*_0_ + Δ at the *σ*_−_ polarization state. This method is normally referred to as Floquet expansion that we mentioned before. We introduce the sum and difference of the density matrix elements as follows: ${\rho }_{s}^{ < 1 > }={\rho }_{11}^{ < 1 > }+{\rho }_{22}^{ < 1 > }$, ${\rho }_{d}^{ < 1 > }={\rho }_{22}^{ < 1 > }-{\rho }_{11}^{ < 1 > }$, ${\sigma }_{s}^{ < 1 > }={\rho }_{31}^{ < 1 > }+{\rho }_{32}^{ < 1 > }$, ${\sigma }_{d}^{ < 1 > }={\rho }_{32}^{ < 1 > }-{\rho }_{31}^{ < 1 > }$, ${\rho }_{s}^{(0)}={\rho }_{11}^{(0)}+{\rho }_{22}^{(0)}$, ${\rho }_{d}^{(0)}={\rho }_{22}^{(0)}-{\rho }_{11}^{(0)}$, ${\sigma }_{s}^{(0)}={\rho }_{13}^{(0)}+{\rho }_{23}^{(0)}$, ${\sigma }_{d}^{(0)}={\rho }_{23}^{(0)}-{\rho }_{13}^{(0)}$. And for a negligible *δ*_*B*_, ${\rho }_{d}^{(0)}$ and ${\sigma }_{d}^{(0)}$ are zeros^31^. The equations for these transformed <1>-labeled Floquet-expansion coefficients can be written as

2 $${\partial }_{t}{\sigma }_{s}^{ < 1 > }=-\,({\rm{\Gamma }}+i{\rm{\Delta }}){\sigma }_{s}^{ < 1 > }-i\frac{\sqrt{2}}{2}(2-3{\rho }_{s}^{(0)}{){\rm{\Omega }}}_{\parallel }+i\frac{3\sqrt{2}}{2}{{\rm{\Omega }}}_{c}{\rho }_{s}^{ < 1 > },$$

3 $${\partial }_{t}{\sigma }_{d}^{ < 1 > }=-\,({\rm{\Gamma }}+i{\rm{\Delta }}){\sigma }_{d}^{ < 1 > }-\frac{\sqrt{2}}{2}(2-3{\rho }_{s}^{(0)}){{\rm{\Omega }}}_{\perp }+i\frac{\sqrt{2}}{2}{{\rm{\Omega }}}_{c}{\rho }_{d}^{ < 1 > },$$

4 $${\partial }_{t}{\rho }_{s}^{ < 1 > }=-\,({{\rm{\Gamma }}}_{0}+{\gamma }_{t}+i{\rm{\Delta }}){\rho }_{s}^{ < 1 > }-i\frac{\sqrt{2}}{2}{\sigma }_{s}^{(0)}{{\rm{\Omega }}}_{\parallel }-\sqrt{2}{{\rm{\Omega }}}_{c}\,\Im {\sigma }_{s}^{ < 1 > },$$

5 $${\partial }_{t}{\rho }_{d}^{ < 1 > }=-\,({\gamma }_{t}+i{\rm{\Delta }}){\rho }_{d}^{ < 1 > }-\,\frac{\sqrt{2}}{2}{\sigma }_{s}^{(0)}{{\rm{\Omega }}}_{\perp }-\,\sqrt{2}{{\rm{\Omega }}}_{c}\,\Im {\sigma }_{d}^{ < 1 > }.$$

Where $\Im$ stands for the imaginary part, *γ*_*t*_ is the population decay rate resulting from the thermal motion, and $\frac{{{\rm{\Gamma }}}_{0}}{2}$ (Γ) denotes the population (optical coherence) decay rate. We assume that ${\rm{\Gamma }}\gg {{\rm{\Gamma }}}_{0}\gg {\delta }_{B}$, and ${\rm{\Gamma }}\gg {\gamma }_{t}\gg {\delta }_{B}$. The detailed explanation on the mechanism of Λ-type CPO can be found in ref.^32^ Here we would like to put a brief discussion here. If the polarization of the probe field is parallel to that of the control field (Ω_⊥_ = 0), then Eq. (3) indicates that ${\sigma }_{d}^{ < 1 > }$ drops to zero quickly due to the large decay rate Γ. And ${\sigma }_{d}^{ < 1 > }=0$ means that the atomic polarizations excited by the probe field on the two optical transitions are of the same amplitude and most importantly, in phase. This leads to the vanishing ${\rho }_{d}^{ < 1 > }$ as well according to Eq. (5). On the contrary, the summation of population on |1〉, |2〉, represented by ${\rho }_{s}^{ < 1 > }$ is oscillating according to Eq. (4). The population oscillates between the ground states (|1〉, |2〉) and the excited state (|3〉) just like a two-level system, and such oscillation decays at the rate of Γ_0_ + *γ*_*t*_.

If the probe and the control field have the orthogonal polarizations (${{\rm{\Omega }}}_{\parallel }=0$). Then Eq. (2) tells us that ${\sigma }_{s}^{ < 1 > }$ tends to zero rapidly, which means that the two probe-field related atomic polarizations on the optical transitions are in *opposite* phase. Then ${\rho }_{s}^{ < 1 > }$ vanishes, meanwhile ${\rho }_{d}^{ < 1 > }$, the difference between the population of the ground levels oscillates, as indicated by Eqs (4) and (5). In other words, two set of closely related, and equal-strength population oscillations take place in opposite phase on transitions |1〉−|3〉 and |2〉−|3〉 (${\sigma }_{s}^{ < 1 > }=0$, ${\rho }_{s}^{ < 1 > }=0$). And it appears like the oscillation happen between the two ground state. And ${\rho }_{d}^{ < 1 > }$ can be regarded as a measure of CPO strength. Such modulation of the population difference opens a narrow transparency window on the absorption profile, and this long-lived oscillation, decaying at the rate of *γ*_*t*_ corresponds to the CPO that we commonly refer to.

From now on, we set ${{\rm{\Omega }}}_{\parallel }=0$, $\Re {{\rm{\Omega }}}_{\perp }=0$, and $\Im {{\rm{\Omega }}}_{\perp }={\rm{\Omega }}$. Here $\Re$ stands for the real part. Then the steady-state solutions are:

6 $$\Re {\sigma }_{d}^{ < 1 > }=\frac{{\rm{\Omega }}{\rm{\Delta }}}{A}[2{\rm{\Gamma }}({\gamma }_{t}^{2}+{{\rm{\Delta }}}^{2})-(3{\rm{\Gamma }}+{\gamma }_{t}){{\rm{\Omega }}}_{c}^{2}],$$

7 $$\Im {\sigma }_{d}^{ < 1 > }=\frac{2{{\rm{\Omega }}{\rm{\Gamma }}}^{2}}{A}({\gamma }_{t}^{2}+{{\rm{\Delta }}}^{2})-\,\frac{{{\rm{\Omega }}{\rm{\Omega }}}_{c}^{2}}{A}({\rm{\Gamma }}{\gamma }_{t}-{{\rm{\Delta }}}^{2}),$$

8 $${\sigma }_{s}^{ < 1 > }={\rho }_{s}^{ < 1 > }=0.$$

**Figure 2:**
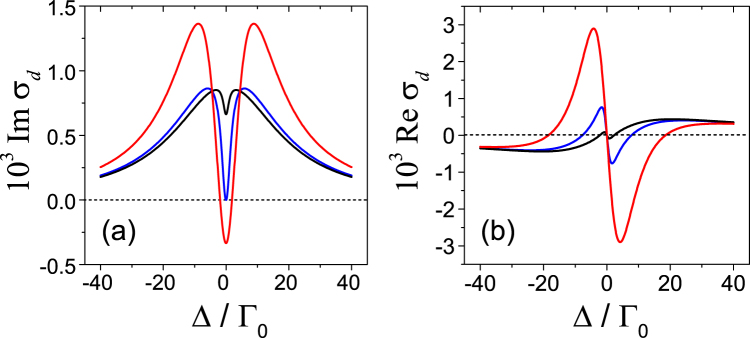
Real part (**a**) and imaginary part (**b**) of *σ*_*d*_. Here $\Im {\sigma }_{d}=0$ when Δ = 0 under the condition of ${{\rm{\Omega }}}_{c}^{2}=2{\gamma }_{r}{\rm{\Gamma }}$ (blue line). The black and red line represent the data for Ω_*c*_ = 2Γ_0_, and 15Γ_0_ respectively. Parameters for the results are: Γ/Γ_0_ = 20, *γ*_*t*_/Γ_0_ = 1, and Ω = 0.1Γ_0_.

Where $A=2\sqrt{2}(1+{s}_{0}){\rm{\Gamma }}[({{\rm{\Gamma }}}^{2}+{{\rm{\Delta }}}^{2})({\gamma }_{t}^{2}+{{\rm{\Delta }}}^{2})+({\rm{\Gamma }}{\gamma }_{t}-{{\rm{\Delta }}}^{2}){{\rm{\Omega }}}_{c}^{2}]$, ${s}_{0}=3{{\rm{\Omega }}}_{c}^{2}/[{\rm{\Gamma }}({\gamma }_{t}+{{\rm{\Gamma }}}_{0})]$. The numerical result of *σ*_*d*_ is given in Fig. 2. $\Im {\sigma }_{d}$ represents the absorption profile with the full width at half maximum as 2Γ approximately. As we can see that, when a relatively weak control field is applied, for example, Ω_*c*_ = 2Γ_0_ (black line in Fig. 2(a)), a transparency window or a dip appears at the center of the absorption line and its minimum can be easily obtained by setting Δ = 0 in Eq. (7).

9 $$\Im \,{\sigma }_{dip}^{ < 1 > }=\frac{{\rm{\Omega }}(2{\gamma }_{t}{\rm{\Gamma }}-{{\rm{\Omega }}}_{c}^{2})}{2\sqrt{2}(1+{s}_{0}){\rm{\Gamma }}({\rm{\Gamma }}{\gamma }_{t}+{{\rm{\Omega }}}_{c}^{2})}.$$

We can see from the above equation that when ${{\rm{\Omega }}}_{c}=\sqrt{2{\gamma }_{t}{\rm{\Gamma }}}$ (blue line in Fig. 2(a)), $\Im {\sigma }_{dip}^{ < 1 > }$ reaches zero. As a matter of fact, we can define a parameter $s={{\rm{\Omega }}}_{c}^{2}/2{\gamma }_{t}{\rm{\Gamma }}$ as the saturation parameter^29^, and in this very situation *s* = 1.

For large control field corresponding to *s* > 1, the red line in Fig. 2(a) shows that the amplification can take place at Δ = 0. To reveal the state of CPO in this case, one has to look into Eqs (3,5) where ${\sigma }_{d}^{ < 1 > }$ and ${\rho }_{d}^{ < 1 > }$ couple with each other. Based on these equations, for the resonant field, we have

10 $${\rho }_{d}^{ < 1 > }=(2-3{\rho }_{s}^{(0)})\frac{{\rm{\Omega }}}{{{\rm{\Omega }}}_{c}+\frac{{\rm{\Gamma }}{\gamma }_{t}}{{{\rm{\Omega }}}_{c}}}.$$

Note that in deriving the above expression, ${\sigma }_{s}^{(0)}$ is disregarded, since it is proportional to $1/{{\rm{\Omega }}}_{c}$. We treat ${\rho }_{d}^{ < 1 > }$ in Eq. (10) as a function of Ω_*c*_ in this paragraph. The term in parentheses relates to the population distribution due to control field solely, and decreases slowly for the increasing Ω_*c*_. The next term in the form of a fraction decreases in the range of ${{\rm{\Omega }}}_{c} > \sqrt{{\rm{\Gamma }}{\gamma }_{t}}$. After all, for ${{\rm{\Omega }}}_{c} > \sqrt{2{\rm{\Gamma }}{\gamma }_{t}}$ or *s* 1, ${\rho }_{d}^{ < 1 > }$ becomes smaller as control field gets stronger since the strong Ω_*c*_ tends to equally distribute the population over the three levels and results in the vanishing population difference. However ${\rho }_{d}^{ < 1 > }$ drives ${\sigma }_{d}^{ < 1 > }$ in Eq. (3) with the coefficient ${{\rm{\Omega }}}_{c}/\sqrt{2}$, see the last term ($i{{\rm{\Omega }}}_{c}{\rho }_{d}^{ < 1 > }/\sqrt{2}$) in Eq. (3). And simple calculations can show that the modulus of ($i{{\rm{\Omega }}}_{c}{\rho }_{d}^{ < 1 > }/\sqrt{2}$) is still a significant value $(\sim \frac{{\rm{\Omega }}}{7\sqrt{2}})$ which notably enlarges $|{\sigma }_{d}^{ < 1 > }|$, and it remains as an increasing function around ${{\rm{\Omega }}}_{c}=\sqrt{2{\rm{\Gamma }}{\gamma }_{t}}$. In conclusion, despite small-amplitude CPO that the larger Ω_*c*_ leads to, the coupling process still increases the polarization, force the atom to radiate, and results in the amplification of the probe field after all.

Next we briefly discuss the relation between the susceptibility *χ* and *σ*_*d*_. As we have set that ${{\rm{\Omega }}}^{\parallel }=0$, and ${{\rm{\Omega }}}^{\perp }=i{\rm{\Omega }}$, consequently ${{\rm{\Omega }}}^{+}=-\,{\rm{\Omega }}/\sqrt{2}$, ${{\rm{\Omega }}}^{-}={\rm{\Omega }}/\sqrt{2}$. The negative sign means phase difference of *π*. Then the relation ${\sigma }_{s}^{ < 1 > }=0$ from Eq. (8) indicates that the susceptibilities for Ω _+_ and Ω_−_ are the same which is reasonable in our model. And it takes form of $\chi =\alpha {\sigma }_{d}^{ < 1 > }/\sqrt{2}{\rm{\Omega }}$, with parameter *α* written as^35^

11 $$\alpha =\frac{N{\wp }^{2}}{{\varepsilon }_{0}\hslash }.$$

Where *N* is the atomic density. With the amplification and attenuation considered, *χ* as well as refractive index *n* become complex numbers^36,37^. When the amplitude of control field changes, *χ* changes accordingly. And this is the fundamental mechanism from which tunable gratings are realized when the standing-wave control field is used.

The CPO system can be realized in cold cesium atoms^38^, helium cell with atoms excited to the metastable state^28,32^, and semiconductor quantum structures^39,40^. Before investigating the features of the standing-wave driven system, we would like to discuss the value of the parameters we have introduced. Here we assume that the helium cell is used to construct the CPO, and the parameters given in the articles^28,32^ are used as references. The transit-population loss rate *γ*_*t*_ results from the thermal motion which simply causes the atom fly out of the range of the laser field. Set *d* as the diameter of Gaussian beam at $1/{e}^{2}$ of maximum intensity, then it can be estimated (Note that *γ*_*t*_ is determined by Gaussian beam diameter (*d*) at $1/{e}^{2}$ of max and the diffusion coefficient (${\mathscr{D}}$) through the relation ${\gamma }_{t}=2{\mathscr{D}}/d$. Where ${\mathscr{D}}=\frac{1}{2}{v}_{m}{\lambda }_{m}$, and ${v}_{m}=\sqrt{8RT/(\pi M)}$ is the mean speed of atoms, ${\lambda }_{m}=RT/(\sqrt{2}\pi {d}_{0}^{2}{N}_{a}P)$ is the mean free path. Here *d*_0_ is the diameter of ^4^He atom, P the pressure of gas, *N*_*a*_ the Avogadro constant, *R* gas constant, *T* the absolute temperature of the gas, and *M* the molar mass. Based on the above relations, for *d* = 1 cm, *γ*_*r*_ is 0.98 KHz) that for *d* = 1 cm, *γ*_*t*_ is approximately 10^3^ rad/s^41^. When the light is strongly focused, for example, *d* = 10 *μ* m, the resulting *γ*_*t*_ is nearly 10^6^ rad/s. Next we consider Γ_0_ which relates to the radiative decay of the atomic level 2^3^*P*, and such radiative decay rate is about 1.022×10^7^ Hz at low enough pressure^42^. Normally in the experiments the pressure is 1 Torr at room temperature^28,32^, leading to the Γ_0_ of megahertz. In our calculation, we assumed that the strongly focused light is used, then the relation *γ*_*t*_/Γ_0_ = 1 is employed.

The optical coherence decay rate Γ relates to the width of absorption profile, hence we need to discuss the Doppler effect first. As already shown in the references^41,43^ the Doppler width *ω*_*D*_ is around 0.85 GHz at the room temperature. And “seen” by the atoms moving at the most probable speed, the frequencies of the forward and backward field (standing-wave control field) are shifted oppositely and the frequency difference acquired is *ω*_*D*_ as well. This shift can be omitted considering a strong control field is used. And the Doppler broadening is taken account of by replacing Γ by *ω*_*D*_. This is reasonable based on the experiments reported in the articles^41,43^. Taking the letter^31^ as the reference, we set Γ/Γ_0_ = 500 in our calculation. The residual Doppler broadening on the spin transition |1〉−|2〉 is simply neglected, since *ρ*_21_ = 0 is previously assumed. The shift *δ*_*B*_ resulting from the Zeeman effect is neglectable as well, for example, 2*δ*_*B*_ = 100 kHz when 17 mG longitudinal magnetic field is used in demonstrating the light storage via CPO^28^. Normally in our system^28^, the control-field Rabi frequency of 28 MHz corresponds to intensity of 198 mW/cm^2^. Then for ${{\rm{\Omega }}}_{c}=\sqrt{2{\gamma }_{t}{\rm{\Gamma }}}$, the control-field intensity is about 0.82 W/cm^2^.

### Properties of the Gratings

In order to construct the spatial periodic variation of the refractive index of the medium, the control field is retro-reflected upon impinging on a mirror of reflectivity *R*_*m*_ to establish a standing-wave pattern within the sample. We explicitly denote the spatial dependence of the Rabi frequency of the control field as Ω_*c*_(*x*), and define it as

12 $${{\rm{\Omega }}}_{c}(x)=\frac{{a}_{m}\sqrt{2{\gamma }_{t}{\rm{\Gamma }}}}{1-\sqrt{{R}_{m}}}\sqrt{{(1+\sqrt{{R}_{m}})}^{2}{\cos }^{2}({k}_{c}x)+{(1-\sqrt{{R}_{m}})}^{2}{\sin }^{2}({k}_{c}x)}.$$

Where *k*_*c*_ = *ω*_*c*_/*c* is the amplitude of the wavevector, and recall that the control field resonant with optical transition, so *ω*_*c*_ = *ω*_0_. Parameter *a*_*m*_ is dimensionless variable representing the amplitude of the control field. Even though the value of *R*_*m*_ is chosen less than unity, we still use “nodes” and “antinodes” to name respectively the positions where the Rabi frequency of the control field takes the minimal and maximal values. At the nodes, ${\min {\rm{\Omega }}}_{c}={{\rm{\Omega }}}_{c}(\frac{\lambda }{4})={a}_{m}\sqrt{2{\gamma }_{t}{\rm{\Gamma }}}$, while at the antinodes ${\max {\rm{\Omega }}}_{c}={{\rm{\Omega }}}_{c}(\frac{\lambda }{2})=\frac{{a}_{m}}{\eta }\sqrt{2{\gamma }_{t}{\rm{\Gamma }}}$, here $\eta =(1-\sqrt{{R}_{m}})/(1+\sqrt{{R}_{m}})$.

One can easily find out that if *a*_*m*_ = *η*, $\Im {\sigma }_{dip}^{ < 1 > }=0$ at antinodes, and if *a*_*m*_ = 1, $\Im {\sigma }_{dip}^{ < 1 > }=0$ at nodes. We can divide the states of the system into three categories regarding the sign of $\Im {\sigma }_{dip}^{ < 1 > }$, as shown in Table 1. We will show that the reflection profiles of the three cases takes the different forms. However, the range of Ω_*c*_ shown in the table can be impractical considering that max Ω_*c*_ should not exceed Γ, and this leads a upper limit as

13 $$\frac{{a}_{m}}{\eta } < \sqrt{\frac{{\rm{\Gamma }}}{2{\gamma }_{t}}}.$$

**Table 1:** The three cases of the standing-wave driven CPO system.

Case	Range of *a*_*m*_	Im ${\sigma }_{d}^{ < 1 > }$ at antinodes	Im ${\sigma }_{d}^{ < 1 > }$ at nodes
I	0 < *a*_*m*_ ≤ *η*	≥0	>0
II	*η* < *a*_*m*_ < 1	<0	>0
III	*a*_*m*_ ≥ 1	<0	≤0

The control field can tailor the optical property of the sample and lead to a spatially periodic refractive index $n=\sqrt{1+\chi }$. In the present CPO system, $|\chi |\ll 1$, then $n=1+\frac{\chi }{2}$. The reflectivity of the probe field can be obtained through characteristic matrix method which is to divide one period (length of ${\lambda }_{c}/2$ here, with *λ*_*c*_ being the wavelength of control field) into *m* layers and treat each layer as a homogeneous dielectric film for a very large *m*. This method is fully studied and can be found in the famous book^44^ and articles^36,37,45^. The spatial period of the grating is half of the probe wavelength, therefore the high-order diffraction cannot be realized in the present atomic configuration, and the probe field should be normal-incident laser. For the transverse-electric polarized light the characteristic matrix for a homogeneous optical layer is

14 $${M}_{j}=(\begin{array}{cc}\cos (k{n}_{j}{d}_{j}) & -\frac{i}{{p}_{j}}\,\sin (k{n}_{j}{d}_{j})\\ -i{p}_{j}\,\sin (k{n}_{j}{d}_{j}) & \cos (k{n}_{j}{d}_{j})\end{array}).$$

Here ${p}_{j}=\sqrt{{\varepsilon }_{j}/{\mu }_{j}}$ with *ε*_*j*_ and *μ*_*j*_ being the dielectric constant and magnetic permeability respectively. Commonly *μ*_*j*_ can be regarded as unity for transparent substances^44^, and *ε*_*j*_ is a complex number^36,37^ whose imaginary part describes absorption or amplification. *k* = *ω*_*p*_/*c*, and *d*_*j*_ = *λ*_*c*_/2*m* is the length of single film. The total characteristic matrix of the sample is

15 $$M={(\prod _{j=1}^{m}{M}_{j})}^{2{\mathscr{N}}}.$$

Here ${\mathscr{N}}=L/{\lambda }_{c}$ is the number of the *control-field wavelength λ*_*c*_ contained by the whole sample (*L*). The reflectivity *R* and transimissivity *T* can be expressed as the function of *m*_*ij*_, the element of *M*.

16 $$R={|\frac{({m}_{11}+{m}_{12}{p}_{l}){p}_{1}-({m}_{21}+{m}_{22}{p}_{l})}{({m}_{11}+{m}_{12}{p}_{l}){p}_{1}+({m}_{21}+{m}_{22}{p}_{l})}|}^{2},$$

17 $$T={|\frac{2{p}_{1}}{({m}_{11}+{m}_{12}{p}_{l}){p}_{1}+({m}_{21}+{m}_{22}{p}_{l})}|}^{2}.$$

Consequently, the absorptivity can be expressed as *A* = 1−*T*−*R*. The parameter ${p}_{1(l)}=\sqrt{{\varepsilon }_{1(l)}/\mu }$ with the subscript 1(*l*) stands for the first (last) layer, which normally is the walls of the container, and we set them as vacuum for simplicity.

### Case I

**Figure 3:**
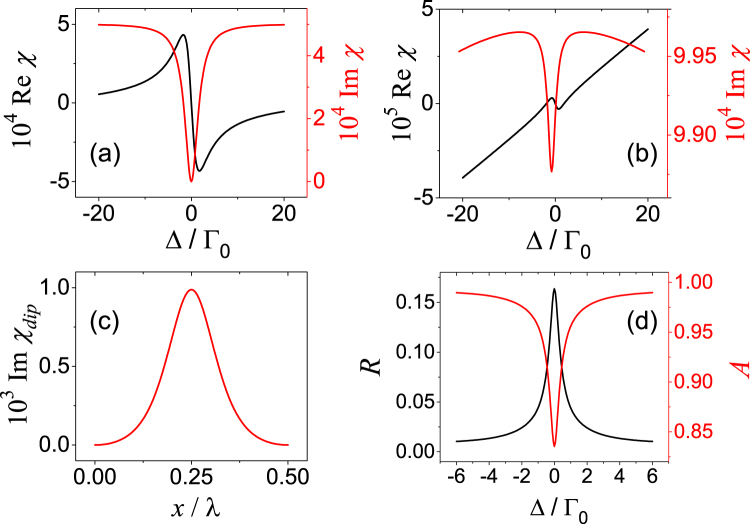
Susceptibility *χ* at antinodes (**a**) and nodes (**b**) for *a*_*m*_ = *η* as the function of probe detuning Δ. (**c**) Imaginary part of the susceptibility for resonant probe field $\Im {\chi }_{dip}$ within a spatial period. Such quantity can represent the modulation on the refractive index due to the relation of $n=1+\chi /2$. (**d**) The reflectivity (red line) and absorptivity (black line) plotted as function of Δ. Parameters are Γ = 500Γ_0_, *γ*_*t*_ = Γ_0_, *λ*_*c*_ = 1.083*μ* m, *L* = 5 mm, *R*_*m*_ = 0.8, *η* = 0.0557, *α* = Γ_0_ and *a*_*m*_ = *η*.

In the first case, the value of *a*_*m*_ locates in $0 < {a}_{m}\le \eta$, the sample is passive at any position. As a special case, *a*_*m*_ = *η* is included in this case and it corresponds to the situation in which zero-absorption happens at antinodes. We present the numerical results of the susceptibility *χ* at antinodes and nodes as Fig. 3(a) and (b) respectively. The special case of *a*_*m*_ = *η* is chosen. As we can see that in Fig. 3(a), the $\Im \chi$ reaches zero in the middle of the dip at the antinodes. Naturally, the absorption is much higher at the nodes due to the weaker the control fields there and the resultant shallower dip is shown in Fig. 3(b).

The susceptibility varies continuously with respect to *x* as shown in Fig. 3(c) where the data of $\Im {\chi }_{dip}$ within one period is plotted. Through out the whole medium, the susceptibility is periodically modified to establish Bragg reflections off the absorption peaks^18^.

Figure 3(d) shows the reflectivity (black line) and the absorptivity (red line) of the system. Naturally, the medium is much absorptive for Δ≠0 than the resonant situations, see Fig. 2(a) and (b). Consequently, it results in the narrow “valley” of the absorption profile. The mirror-symmetry of the absorptivity and reflectivity profile suggests that the absorption limits the reflection, and shapes the reflection profile. For the even more weak control field, *a*_*m*_ < *η*, the medium at the antinodes is absorptive, then the reflection will be even weak. The low reflectivity certainly limits the application of the system, however the situation changes in the next case.

### Case II

**Figure 4:**
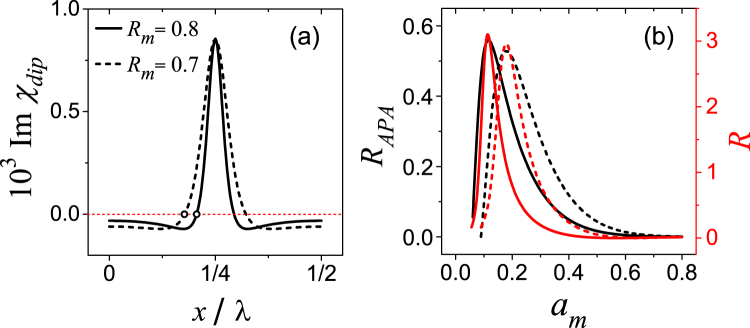
(**a**) The imaginary part of *χ*_*dip*_ under different values of *R*_*m*_. The small circles indicate the positions of *x*_0_. The data is calculated using Eqs (9) and (12) with *a*_*m*_ = 0.2; (**b**) Relation between reflectivity and the amplitude of control field. Different linecolors distinguish the reflectivity obtained from the two methods: Black lines indicate that the data is calculated using the APA approximation method, while the data represented by red lines is obtained from Eq. (16). Two kinds of lineshapes are used to differentiate values of *R*_*m*_: solid line for *R*_*m*_ = 0.8, and dashed line for *R*_*m*_ = 0.7. Other parameters are *α* = Γ_0_, Γ = 500Γ_0_, *γ*_*t*_ = Γ_0_, *λ*_*c*_ = 1.083*μ* m, *L* = 5 mm, and Δ = 0.

The larger control field can lead to the amplification of the probe field. In this subsection we discuss the control field whose amplitude is under the condition of *η* < *a*_*m*_ < 1. In this case, $\Im {\chi }_{dip} < 0$ (amplification) at the antinodes, meanwhile $\Im {\chi }_{dip} > 0$ (attenuation) at nodes, as shown in Fig. 4(a) where $\Im {\chi }_{dip}$ is plotted as a function of *x* for *R*_*m*_ = 0.7 (dashed line) and *R*_*m*_ = 0.8 (solid line). The zero point which is marked by the small circle is set to be (*x*_0_,0), and *x*_0_ can be obtained from Eq. (7) which is

18 $${x}_{0}=\frac{\lambda }{4\pi }\arccos [\frac{{(1-\sqrt{{R}_{m}})}^{2}-{a}_{m}^{2}(1+{R}_{m})}{2{a}_{m}^{2}\sqrt{{R}_{m}}}].$$

Based on the sign of $\Im {\chi }_{dip}$, a single spatial period of the standing-wave control field can be roughly and rationally divided into three layers which are the first active layer $0\le x\le {x}_{0}$, the second passive layer ${x}_{0} < x < {\lambda }_{c}/2-{x}_{0}$, and the last active layer $\frac{{\lambda }_{c}}{2}-{x}_{0}\le x\le \frac{{\lambda }_{c}}{2}$. This simplified active-passive-active (APA) three-layer system is featured by the thickness of each layer which relates to *x*_0_, and averaged dielectric constant of each layer. Note that from the angle of averaged dielectric constant, the first and the third layers are identical. Here we introduce parameter ${\bar{\varepsilon }}_{a(p)}$ to represent the averaged dielectric constant of the active (passive) layer, and it takes the form of ${\bar{\varepsilon }}_{a(p)}=1+{\bar{\chi }}_{a(p)}+i{\bar{\kappa }}_{a(p)}$, with

19 $${\bar{\kappa }}_{a}=\frac{\alpha }{{x}_{0}}{\int }_{0}^{{x}_{0}}\frac{\Im {\sigma }_{dip}^{ < 1 > }(x)}{\sqrt{2}{\rm{\Omega }}}dx,$$

20 $${\bar{\kappa }}_{p}=\frac{\alpha }{{\lambda }_{c}/4-{x}_{0}}{\int }_{{x}_{0}}^{{\lambda }_{c}\mathrm{/4}}\frac{\Im \,{\sigma }_{dip}^{ < 1 > }(x)}{\sqrt{2}{\rm{\Omega }}}dx.$$

The expression of *α* is given in Eq. (11). The quantity of ${\bar{\chi }}_{a(p)}$ is obtained through the averaging process of $\Re {\sigma }_{dip}^{ < 1 > }$. However, considering that it is the resonant probe field that we investigate here, ${\bar{\chi }}_{a(p)}$ vanishes. Incidentally, the real and imaginary parts of complex refractive index^46^ correspond to ${\bar{\varepsilon }}_{a(p)}$ can be written as ${(\Re {n}_{a(p)})}^{2}=(\sqrt{1+{\bar{\kappa }}_{a(p)}}+1)/2$, ${(\Im {n}_{a(p)})}^{2}=(\sqrt{1+{\bar{\kappa }}_{a(p)}}-1)/2$. And more information about the approximate method for reflection from the continuously varying refractive index can be found in ref.^45^ After define *θ* = 2*πx*_0_/*λ*, the reflectivity of the APA system is

21 $${R}_{APA}={|\frac{\sin (2\theta )}{2\theta }\frac{2{{\mathscr{N}}}_{\theta }({\bar{\kappa }}_{p}-{\bar{\kappa }}_{a})}{\mathrm{(2}+{{\mathscr{N}}}_{\theta }{\bar{\kappa }}_{a})[2+{{\mathscr{N}}}_{\theta }(\frac{\pi }{2\theta }-1){\bar{\kappa }}_{p}]}|}^{2}.$$

Where ${{\mathscr{N}}}_{\theta }=4\theta {\mathscr{N}}$ and $|{\kappa }_{a(p)}|\ll 1$ is already assumed. The detailed derivation of the formula is presented in the Supplementary Information.

Figure 4(b) provides results of *R* from Eq. (16) (red lines) and *R*_*APA*_ (black lines) for *R*_*m*_ = 0.8 (solid lines) and 0.7 (dashed lines). Clearly, the active medium can lead to the large reflectivity exceeding unity^17^, see the red line in Fig. 4(b). As we can see that for the same value of *a*_*m*_, the formula of *R* provides a relatively larger value of reflectivity than the APA method. This is mainly because that *R*_*APA*_ only takes account of reflection between the layers ${\bar{\varepsilon }}_{p}$ and ${\bar{\varepsilon }}_{a}$. The variation of the refraction index within each APA-layer is averaged, the corresponding reflection is neglected as well. However, the similarity of the line profile of *R* and *R*_*APA*_ as functions of *a*_*m*_ is very high, especially on the value of *a*_*m*_ for which the maximum reflection takes place. And such value is determined by ${\bar{\varepsilon }}_{a}$, ${\bar{\varepsilon }}_{p}$ and *x*_0_ through Eq. (21). Clearly, the APA structure is main factor of the reflection profile. The reflection from the interface of the passive and active layers dominates the reflection feature due to its more significant variation of the optical properties. And the formula (21) can be employed as a quick reference for controlling the reflection profile (more precisely, the relative magnitude of reflection) using the coupling-field intensity, however, it is unquestionably the rough estimation on the certain value of the reflectivity.

**Figure 5:**
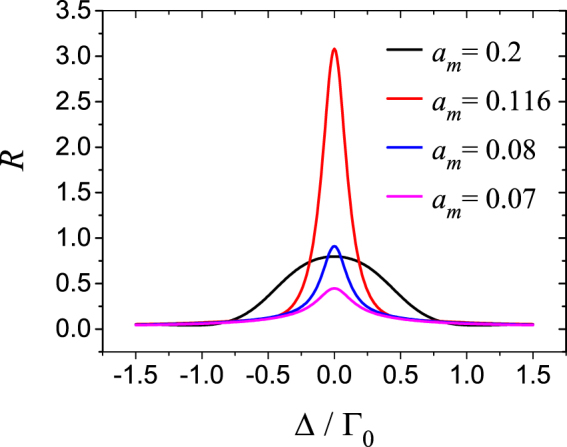
(**a**) The reflectivity plotted as a function of probe detuning under different values of *a*_*m*_. *R*_*m*_ = 0.8; Other parameters are *α* = Γ_0_, Γ = 500Γ_0_, *γ*_*t*_ = Γ_0_, *λ*_*c*_ = 1.083*μ* m, *L* = 5 mm.

The reflectivity provided in Fig. 4(b) is for the resonant probe field. In Fig. 5 we present the reflectivity *R* as the function of Δ, under four different values of *a*_*m*_. Together with Fig. 4(b), Fig. 5 shows how the reflectivity can be controlled by the amplitude of the control field. The red line corresponds to *a*_*m*_ = 0.116, and such value leads to the maximum reflection in Fig. 4(b) for *R*_*m*_ = 0.8; In the medium contains the gain layers, the reflectivity could exceed unity just as we shown here. For the other value of *a*_*m*_, the different structure of APA layers are constructed, the probe field somehow does not resonate with the gratings anymore, and lower reflectivity is caused. Note that for the stronger control field, the width of the reflection peak is larger. This is simply because that more atoms within the Doppler broadening is involved in the pumping process.

### Case III

In this subsection we consider the relatively large control field, *a*_*m*_ ≥ 1. The case of *a*_*m*_ ≥ 1 means $\Im {\chi }_{dip}$ is negative everywhere. The “=” sign corresponds to the situation in which $\Im {\chi }_{dip}=0$ at nodes. This case can be difficult to realize due to the limitation of the laser intensity imposed by the capacity of practical device or the requirement of CPO pump mechanics, such as Eq. (13). For the latter one, if we set *R*_*m*_ = 0.8, the Eq. (13) requires *a*_*m*_ < 0.88 and Case III becomes an unreasonable situation; Meanwhile for *R*_*m*_ = 0.7, *a*_*m*_ < 1.4 is demanded. In the following investigation, we set *R*_*m*_ = 0.7, *a*_*m*_ = 1.

**Figure 6:**
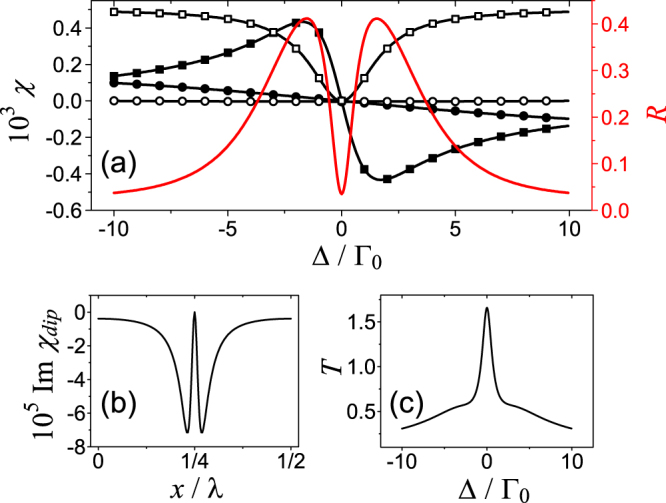
(**a**) The real (solid squares) and imaginary part (open squares) of *χ* at the nodes, together with real (solid circles) and imaginary part (open circles) of *χ* at the antinodes are plotted as the functions of probe detuning. The reflectivity (R) is presented in (**a**) as well using the red-color line. Here *a*_*m*_ = 1.0 and *R*_*m*_ = 0.7. The resultant imaginary part of susceptibility at the dip for different positions is present in (**b**), while the corresponding real part is zero. In (**c**) we provide the numerical result of spectral transmissivity. Other parameters are *α* = Γ_0_, Γ = 500Γ_0_, *γ*_*t*_ = Γ_0_, *λ*_*c*_ = 1.083*μ* m, *L* = 5 mm.

In Fig. 6(a) we present data of $\Re \chi$, $\Im \chi$, and *R* for varying Δ. In contrast with the previous case, here the reflectivity is quite low for the resonant probe field. This is because that $\Im {\chi }_{dip}$ varies over a very narrow range within a spatial period as shown in Fig. 6(b), and $\Re {\chi }_{dip}$ is a constant just as in the previous cases. Note that in Fig. 6(b) the data is magnified 10^5^ times.

As for the amplification, $\Im {\chi }_{dip}$ is nearly negative everywhere, which means the whole sample can be regarded as the gain medium for the resonant probe field. This is feature of the current case. However, the spatial deviation of the susceptibility is so small, and results in the insignificant reflection even amplification is included. And the gain characteristic of the medium manifests itself in the transmission as shown in Fig. 6(c) that *T* exceeds unity for Δ = 0.

The two peaks of the reflectivity appears where the refractive index at the nodes reaches the extreme values. As we can see in Fig. 2(b) that the probe detuning corresponding to the extreme values (Δ_*m*_) does not change much for different values of control-field Rabi frequency near ${{\rm{\Omega }}}_{c}=\sqrt{2{\gamma }_{t}{\rm{\Gamma }}}$. They are around certain values, Δ_*m*_ = ±Δ_0_ we assumed. By solving ${\partial }_{{\rm{\Delta }}}\Im {\sigma }_{d}({\rm{\Delta }})=0$, we have ${{\rm{\Delta }}}_{0}=\sqrt{3}{\gamma }_{t}$ approximately under *s* = 1. The reason for such insensitive response to Ω_*c*_ is the width of the dip which relates to *γ*_*t*_ solely and the determinative connection between the $\Re \chi$ and $\Im \chi$ through Kramers-Kronig relations^47^. For the larger coupling-field Rabi frequency far from ${{\rm{\Omega }}}_{c}=\sqrt{2{\gamma }_{t}{\rm{\Gamma }}}$, the detuning for maximal refractive index drift away from ±Δ_0_. However, the much smaller refractive index is caused as well, see $\Re \chi$ at the antinodes represented by the lines with solid circles in Fig. 6(a). As the intensity of the control field changes through the spatial period, those extreme refractive indices apparently change most significantly, and result in the two peaks of the reflectivity.

The reflectivity could be enhanced by increasing the atomic density *N* due to the enlarged spatial deviation of the complex refractive index and the enhanced gain feature. However the latter one could cause the probe field (either reflected or transmitting) to “blow” up, and such instability is not helpful in practical application. The intensity of control field is relatively high in this case, for example, the data used in Fig. 6 requires the control intensity at the antinodes to be approximately 103.7 W/cm^2^. This requirement depends on Γ, and cooling the sample to reduce the Doppler broadening could makes the lower control-field intensity valid in this case. Comparing the results in the previous case, the situation here is *not* a reasonable choice for controlling the reflectivity in practice. However it is attractive only when this special reflection line-profile is demanded.

In all the three cases, we assumed that the coupling field resonates with the optical transition. For the non-resonant coupling field, if the coupling detuning is smaller than Γ, spectral reflectivity as a function of Δ should not change much since the CPO depends on Δ, the frequency difference of the coupling and probe field^29^. The period of the grating can be further tuned through the misaligning angle between the two component lasers of the standing-wave coupling field^37^. The period increases with the misaligning angle, and causes the reflection profile to shift due to the Bragg’s rule.

## Conclusions

In this paper, we investigate the optically controllable gratings in the CPO medium whose refractive index can be periodically modulated by the standing-wave control field. The valuable feature of CPO is the dip appears in the absorption profile with the width relating to the population decay rate and the depth depending on the strength of the control field. When the Rabi frequency of the control field is larger than the saturation parameter, the medium becomes active for the resonant probe field, and amplification appears. We use the standing wave as the control field, then the dip varies spatially to form a standing-wave pattern. The resultant Bragg scattering can lead to significant reflection. Distinguished by the control-field intensity and the reflectivity of cavity mirror represented by *R*_*m*_ or *η* in the text, the medium is divided into three categories regarding absorption property of medium (see Tab. 0). The lower control-field intensity makes the medium absorptive at any position. In such case the reflection profile is characterized by one sharp peak locating in the resonance region. The reflectivity is low due to the limitation imposed by the attenuation process. The next case relates to the stronger control field which forces the medium to be active at anti-nodes meanwhile still passive at nodes for the resonance field. With the aid of the simplified three-layer APA approximate method, we show that how amplification changes the scattering property of the medium thoroughly. The most significant feature is that the amplification can lead to the reflection profile with one peak larger than unity. In addition, it can provide a flexible way to control the reflectivity through the control field as shown in Fig. 5. Although it is difficult to realize practically, for the complete analysis, we consider the third case as well in which the even stronger control field is assumed, and it makes absorption disappears. As an example, we investigate the critical state in which the medium is neither gain nor absorptive at nodes, but active everywhere else. The spectral reflectivity has two peaks whose locations depend on the population decay rate between the ground states. The resonant field can propagate through the medium and get amplified, but barely reflected due to the small variation of the complex refractive index. At last we would like to mention that the present system can be extended. Using a standing-wave coupling field and a traveling-wave coupling field to form a two-photon transition^48,49^, a subwavelength grating could be constructed.

### Data Availability

No datasets were generated or analysed during the current study.

## Electronic supplementary material

Supplementary material
