# Supplementary material for: Optically Tunable Gratings Based on Coherent Population Oscillation
Source: Sci Rep. 2018 May 1;8:6834. doi: 10.1038/s41598-018-25010-w (PMC5931600; doi:10.1038/s41598-018-25010-w)
Supplement: Supplementary file 1 — Supplementary material [file 41598_2018_25010_MOESM1_ESM.pdf]

Supplementary material for "Optically Tunable Gratings Based on Coherent Population Oscillation"

Xiao-Jun Zhang[1,2], Hai-Hua Wang[3], Lei Wang[3], and Jin-Hui Wu[1,*]

[1] *Center for Quantum Sciences, Northeast Normal University, Jingyue Street 2555, Changchun 130117, China*
[2] *Changchun Observatory, National Astronomical Observatories, CAS, Changchun 130117, China*
[3] *College of Physics, Jilin University, Changchun 130023, China*
[*] *jhwu@nenu.edu.cn*

1 Density matrix equations

The zero-order equations are

$$\partial_t \rho_{32}^{(0)} = (i\delta_B - \Gamma)\,\rho_{32}^{(0)} + i\frac{\Omega_c}{\sqrt{2}}\left(\rho_{22}^{(0)} - \rho_{33}^{(0)}\right), \tag{1}$$

$$\partial_t \rho_{31}^{(0)} = (-i\delta_B - \Gamma)\,\rho_{31}^{(0)} + i\frac{\Omega_c}{\sqrt{2}}\left(\rho_{11}^{(0)} - \rho_{33}^{(0)}\right), \tag{2}$$

$$\partial_t \rho_{22}^{(0)} = \frac{\gamma_t}{2} + \frac{\Gamma_0 \rho_{33}^{(0)}}{2} - \gamma_t \rho_{22}^{(0)} + i\frac{\Omega_c}{\sqrt{2}}\Re(\rho_{32}^{(0)}), \tag{3}$$

$$\partial_t \rho_{11}^{(0)} = \frac{\gamma_t}{2} + \frac{\Gamma_0 \rho_{33}^{(0)}}{2} - \gamma_t \rho_{11}^{(0)} + i\frac{\Omega_c}{\sqrt{2}}\Re(\rho_{31}^{(0)}), \tag{4}$$

$$\rho_{11}^{(0)} + \rho_{22}^{(0)} + \rho_{33}^{(0)} = 1. \tag{5}$$

Here γ_t, Γ_0, and Γ are described in the text. The steady-state solution can be written as

$$\rho_{32}^{(0)} = -\frac{(\delta_B - i\Gamma)\Omega_c}{2\sqrt{2}\,(\delta_B^2 + (1+s_0)\Gamma^2)}, \tag{6}$$

$$\rho_{31}^{(0)} = \frac{(\delta_B + i\Gamma)\Omega_c}{2\sqrt{2}\,(\delta_B^2 + (1+s_0)\Gamma^2)}, \tag{7}$$

$$\rho_{11}^{(0)} = \rho_{22}^{(0)} = \frac{\delta_B^2 + (1+\frac{2}{3}s_0)\Gamma^2}{2\delta_B^2 + 2(1+s_0)\Gamma^2}. \tag{8}$$

Here $s_0 = 3\Omega_c^2/\left[\Gamma\left(\gamma_t + \Gamma_0\right)\right]$. As we can see that from the Eq. (8), for a strong control field, the population is equally distributed on the three atomic levels. The first-order equations are:

$$\partial_t \rho_{32}^{(1)} = (i\delta_B - \Gamma)\,\rho_{32}^{(1)} + i\Omega_{c-}\left(\rho_{22}^{(1)} - \rho_{33}^{(1)}\right) + i\Omega_- e^{i\Delta t}\left(\rho_{22}^{(0)} - \rho_{33}^{(0)}\right), \tag{9}$$

$$\partial_t \rho_{31}^{(1)} = -\,(i\delta_B + \Gamma)\,\rho_{31}^{(1)} + i\Omega_{c+}\left(\rho_{11}^{(1)} - \rho_{33}^{(1)}\right) + i\Omega_+ e^{i\Delta t}\left(\rho_{11}^{(0)} - \rho_{33}^{(0)}\right), \tag{10}$$

$$\partial_t \rho_{22}^{(1)} = -\gamma_t \rho_{22}^{(1)} - \frac{1}{2}\Gamma_0(\rho_{11}^{(1)} + \rho_{22}^{(1)}) - i\rho_{23}^{(1)}\Omega_{c-} + i\rho_{32}^{(1)}\Omega_{c-} - ie^{i\Delta t}\rho_{23}^{(0)}\Omega_- + ie^{-i\Delta t}\rho_{32}^{(0)}\Omega_-^*, \tag{11}$$

$$\partial_t \rho_{11}^{(1)} = -\gamma_t \rho_{11}^{(1)} - \frac{1}{2}\Gamma_0(\rho_{11}^{(1)} + \rho_{22}^{(1)}) - i\rho_{13}^{(1)}\Omega_{c+} + i\rho_{31}^{(1)}\Omega_{c+} - ie^{i\Delta t}\rho_{13}^{(0)}\Omega_+ + ie^{-i\Delta t}\rho_{31}^{(0)}\Omega_+^*, \tag{12}$$

$$\rho_{11}^{(1)} + \rho_{22}^{(1)} + \rho_{33}^{(1)} = 0. \tag{13}$$

2 The three-layer method

The characteristic matrix of the j-th layer is

$$M^{[j]} = \begin{pmatrix} \cos(k_0 n_j d_j) & -i n_j^{-1} \sin(k_0 n_j d_j) \\ -i n_j \sin(k_0 n_j d_j) & \cos(k_0 n_j d_j) . \end{pmatrix} \tag{14}$$

with n_j being the complex refractive index [1, 2], and d_j the thickness of the layer, $k_0 = \frac{\omega}{c} = \frac{2\pi}{\lambda}$ is the (probe) wavevector in vacuum. The situation which interests us is the three-layer system with complex refractive index $n_j = \sqrt{1 + i\kappa_j}$ where κ_j describes the absorption or amplification. Assume that $|\kappa_j| \ll 1$, we have $n_j = 1 + \frac{i}{2}\kappa_j$. Let us define a matrix $\mathscr{M}$ depending on three parameters G, P, and Q as

$$\mathscr{M}(G, P, Q) = \begin{pmatrix} G & P + Q \\ P - Q & G \end{pmatrix} . \tag{15}$$

Then, to the first order of κ_j, the matrix $M^{[j]}$ can be simplified as $\hat{M}^{[j]} = \mathscr{M}\left(G^{[j]}, P^{[j]}, Q^{[j]}\right)$, with

$$G^{[j]} = \cos\theta - \frac{i}{2}\theta\kappa_j \sin\theta, \tag{16a}$$

$$P^{[j]} = \frac{\kappa_j}{2}\theta\cos\theta - i\sin\theta, \tag{16b}$$

$$Q^{[j]} = -\frac{\kappa_j}{2}\sin\theta. \tag{16c}$$

Let λ_c be the wavelength of the control field. For providing a better analogy of standing-wave coupled CPO system, we set $d_1 + d_2 + d_3 = \frac{\lambda_c}{2}$, $d_1 = d_3$, $\kappa_1 = \kappa_3 = \kappa_a$, and $\kappa_2 = \kappa_p$. After defining a new parameter $\theta_1 = \frac{2\pi d_1}{\lambda}$, the characteristic matrix for a single period (three-layer) is $M_{1/2} = \hat{M}^{[1]}\hat{M}^{[2]}\hat{M}^{[3]}$, and the characteristic matrix corresponding to the length of control-field wavelength is $M_1 = \left(M_{1/2}\right)^2 = \mathscr{M}(G_1, P_1, Q_1)$

$$G_1 = 1, \tag{17a}$$

$$P_1 = 2\pi\left(\frac{\kappa_p}{2} + \frac{\Delta\kappa\theta_1}{\pi}\right), \tag{17b}$$

$$Q_1 = -\Delta\kappa \sin(2\theta_1) . \tag{17c}$$

where $\Delta\kappa = \kappa_a - \kappa_p$. Considering such periodically stratified media of the length $(\mathcal{N}\lambda_c)$, the characteristic matrix is

$$M_{\mathcal{N}} = (M_1)^{\mathcal{N}} . \tag{18}$$

If the power of the matrix is performed by keeping the terms on the lowest order of κ_a or κ_p in each element of the matrix, then $M_{\mathcal{N}}$ is reduced to $\mathcal{M}_{\mathcal{N}}$, which is

$$\mathcal{M}_{\mathcal{N}} = \mathscr{M}(G_{\mathcal{N}}, P_{\mathcal{N}}, Q_{\mathcal{N}}) . \tag{19}$$

With

$$G_{\mathcal{N}} = 1 + \frac{1}{2}\mathcal{N}(\mathcal{N} - 1)\left(P_1^2 - Q_1^2\right), \tag{20a}$$

$$P_{\mathcal{N}} = \mathcal{N}P_1, \tag{20b}$$

$$Q_{\mathcal{N}} = \mathcal{N}Q_1. \tag{20c}$$

And this requires

$$\left|P_1^2 - Q_1^2\right| \ll \frac{2\mathcal{N}}{(\mathcal{N} - 1)(\mathcal{N} - 2)}. \tag{21}$$

The reflection coefficient can be calculated by the characteristic matrix form the formula (16) in the text. In Fig. 1(a), we plot the reflectivities calculated from three characteristic matrices, they are

$$R_0 = \left| r\left(\left[\hat{M}^{[1]}\hat{M}^{[2]}\hat{M}^{[3]}\right]^{2\mathcal{N}}\right)\right|^2, \tag{22a}$$

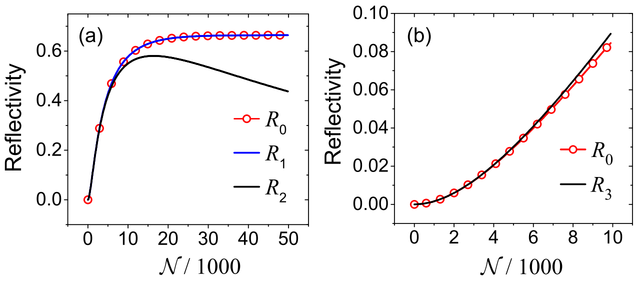

Figure 1: (a) Numerical results of R_0, R_1, and R_2 for the varying $\mathcal{N}$. $\kappa_a = -4 \times 10^{-5}$, $\kappa_p = 4 \times 10^{-4}$. (b) Comparing R_0 and R_3 with smaller value of κ_a and κ_p: $\kappa_a = -4 \times 10^{-6}$, $\kappa_p = 4 \times 10^{-5}$. And $d_1/\lambda = 0.15$ for both figures.

$$R_1 = \left| r \left(M_{\mathcal{N}} \right) \right|^2, \tag{22b}$$

$$R_2 = \left| r \left(\mathcal{M}_{\mathcal{N}} \right) \right|^2. \tag{22c}$$

As we can see that the simplified reflectivity R_1 matches R_0 perfectly, while more deeply simplified value R_2 is reliable for smaller $\mathcal{N}$ due to the condition (21). And R_2 can be written as

$$R_2 = \left| \frac{2\mathcal{N}Q1}{1 + \mathcal{N}P_1 + \frac{\mathcal{N}(\mathcal{N}-1)}{2}\left(P_1^2 - Q_1^2\right)} \right|^2. \tag{23}$$

Substitute Eqs. (17) into the above equation, and neglect the small terms, we have a more compact expression R_3 as

$$R_3 = \left| \frac{\sin(2\theta_1)}{2\theta_1} \frac{2\mathcal{N}_\theta(\kappa_p - \kappa_a)}{(2 + \mathcal{N}_\theta \kappa_a)\left[2 + \mathcal{N}_\theta \left(\frac{\pi}{2\theta_1} - 1\right)\kappa_p\right]} \right|^2. \tag{24}$$

where $\mathcal{N}_\theta = 4\theta_1\mathcal{N}$. We compare the R_0 and R_3 in Fig. 1(b). As an even more simplified expression with $\kappa_{a(p)}$, the validity of R_3 requires much stronger limitation imposed by Eq. (21), as we can see that R_0 and R_3 match each other for even smaller $\mathcal{N}$. The parameters of the CPO system we investigate make R_3 quite suitable to represent the reflectivity of the simplified three-layer APA model. For example, in Fig. 4(b) of the text, the maximal point of red solid line corresponds to $\kappa_a = -8.6 \times 10^{-6}$, and $\kappa_p = 3.2 \times 10^{-4}$. With $\mathcal{N}$ is about 4617, simple calculation can show that they satisfy Eq. (21) very well.

References

[1] M. Artoni, G. La Rocca, and F. Bassani. Resonantly absorbing one-dimensional photonic crystals. *Phys. Rev. E*, 72:046604, Oct 2005.

[2] M. Artoni and G. C. La Rocca. Optically tunable photonic stop bands in homogeneous absorbing media. *Phys. Rev. Lett.*, 96:073905, Feb 2006.
